# Heart transplant for a patient with left superior vena cava: Case report and surgical technique

**DOI:** 10.1016/j.xjtc.2024.07.015

**Published:** 2024-07-30

**Authors:** Mohammad Alomari, Pankaj Garg, Nafiye Busra Celik, J. Ross Renew, Basar Sareyyupoglu

**Affiliations:** a Department of Cardiothoracic Surgery, Mayo Clinic, Jacksonville, Fla; b Department of Anesthesiology and Perioperative Medicine, Mayo Clinic, Jacksonville, Fla

**Figure:**
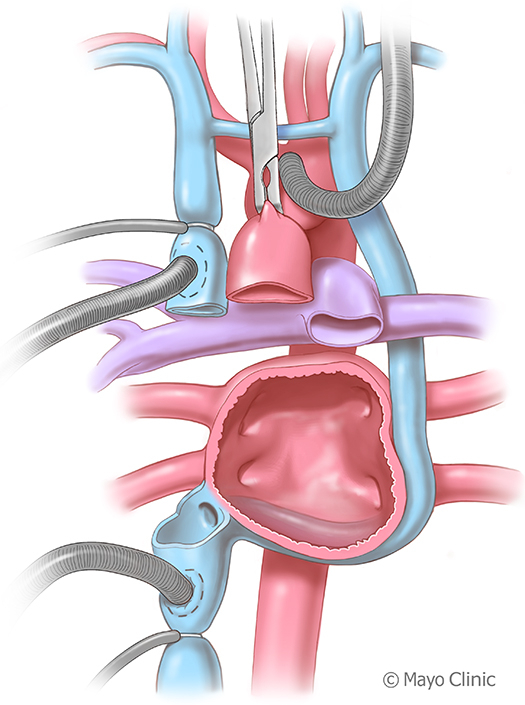
Cardiectomy technique in case of persistent left superior vena cava.

**Central Message**

Our technique for preserving the recipient's coronary sinus and in situ routing the PLSVC by modified cardiectomy and bicaval HTx is simple, reproducible, and avoids prosthetic materials.

The presence of persistent left superior vena cava (PLSVC) in the donor or the recipient is rarely encountered in adult patients undergoing heart transplantation (HTx).^1^, ^2^, ^3^ This report delineates the surgical technique used to route bilateral superior vena cava (SVC) with PLSVC draining into the roofed coronary sinus (CS) concomitant in a recipient undergoing combined heart and kidney transplantation. Institutional review board approval was not required; the patient provided written informed consent for publication of study data.

## Case Presentation

A 68-year-old male patient with a medical history of diabetes mellites, stage III chronic kidney disease, and ischemic cardiomyopathy status post-multiple percutaneous coronary interventions, HeartMate 3 left ventricular assist device (LVAD) implantation in 2018 without complications, and cardiac resynchronization therapy with defibrillator presented for HTx evaluation in April 2023 as the result of progressive right heart failure. His computed tomography of the chest revealed bilateral SVC with small communicating vein. Contrast echocardiography with agitated saline through left arm confirmed the PLSVC was draining into the right atrium (RA) via roofed CS (Video 1). A suitable donor for combined heart and kidney transplantation became available in July 2023.

## Technique

During surgery, uneventful redo sternotomy was performed, and the heart was mobilized by blunt and sharp dissections. After heparinization, cardiopulmonary bypass (CPB) was initiated with cannulation of the ascending aorta, right SVC, and inferior vena cava (IVC). The aorta was crossclamped, the right SVC and IVC caval snares were tightened, the LVAD outflow graft was clamped, and the LVAD was turned off. The aorta and pulmonary artery were divided just distal to their respective valves. The right SVC was transected at the cavoatrial junction. A large cuff of IVC and CS was prepared by transecting the RA adjacent to the IVC and carrying it medially above instead of through the ostium of CS and laterally through the floor of the fossa ovalis. The left atrial cuff with CS was prepared by the development of the interatrial groove and extending the incision along the base of posterior mitral annulus, leaving behind a generous cuff of left atrial tissue, with care taken not to injure the CS. The recipient cardiectomy was completed with concurrent explantation of the LVAD pump. Finally, the left atrial appendage was excised, and the integrity of CS and the orifices of 4 pulmonary veins was confirmed.

**Figure 1:**
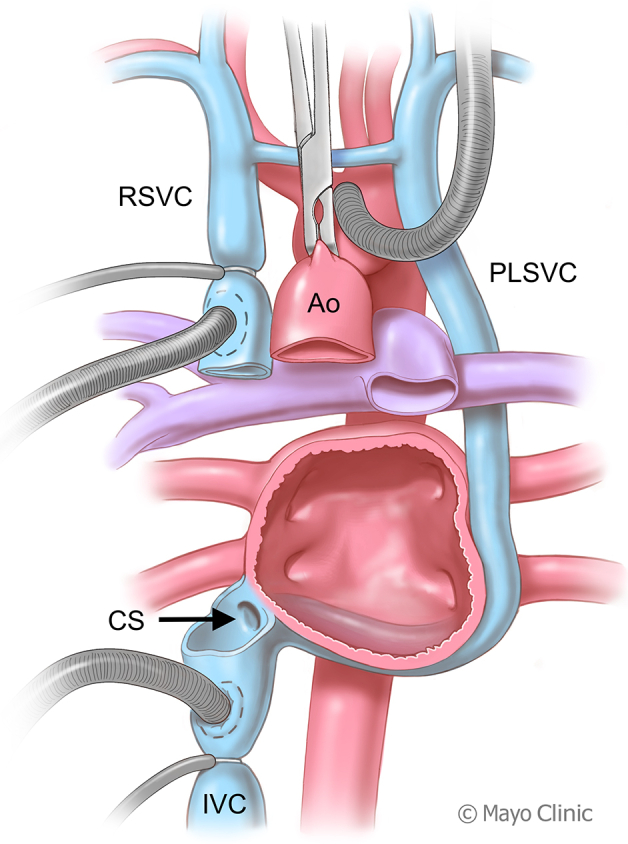
An illustration depicts the cardiectomy technique employed during a bicaval heart transplantation for a recipient with a PLSVC. Cardiectomy was performed, leaving the PLSVC and CS intact and making a common island of IVC and CS. *PLSVC*, Persistent left superior vena cava; *CS*, coronary sinus; *IVC*, inferior vena cava; *RSVC*, right superior vena cava; *Ao*, aorta.

**Figure 2:**
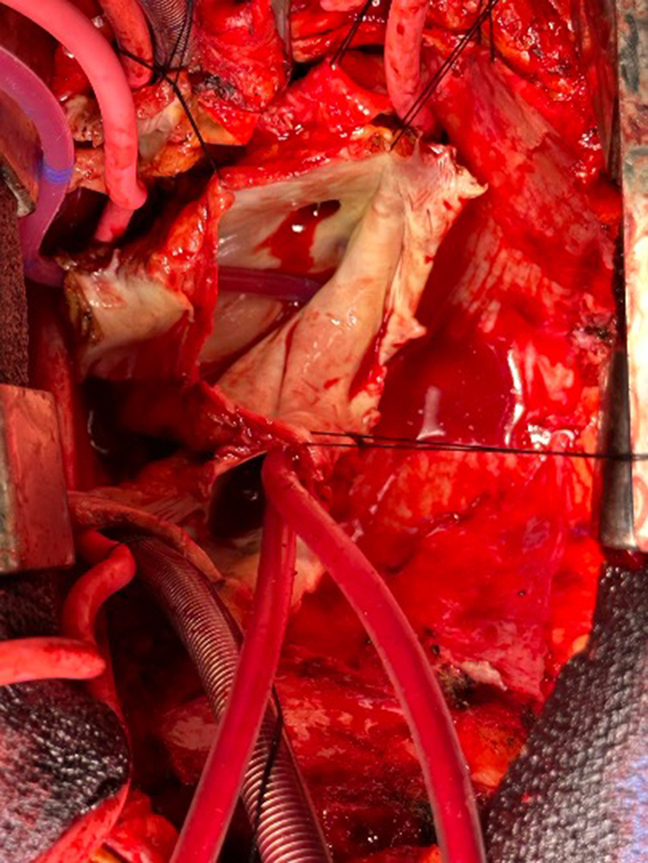
Intraoperative image demonstrated an intact, roofed coronary sinus after cardiectomy. The recipient coronary sinus and inferior vena cava were prepared as a single conduit.

Standard orthotopic HTx with a bicaval technique was performed with several modifications. (1) The PLSVC remained unsnared during cardiectomy to distend and better visualize the CS. Subsequently, the PLSVC was allowed to drain into a cardiotomy sucker placed through the CS opening. (2) The recipient CS opening and IVC were prepared as a unified structure during cardiectomy (Figures 1 and 2). (3) The recipient's left atrioventricular groove remained intact, preserving a cuff of the mitral annulus. (4) The recipient's CS opening and IVC were anastomosed en bloc to the donor's IVC.

After HTx, the patient was weaned off CPB uneventfully. Subsequently, cardiac resynchronization therapy with defibrillator leads and generator was removed, hemostasis was achieved, and the sternum was closed. A kidney transplant was then performed. The postoperative course was unremarkable, and patient was discharged 25 days after the operation. Postoperative echocardiography revealed good biventricular function, the presence of 2 distinct CS in the right atrium, and unobstructed PLSVC drainage (Video 2). At the last follow-up, 9 months later, the patient remained in good health.

## Discussion

The presence of PLSVC in the recipient significantly complicates HTx. Conventional management techniques for PLSVC involve the placement of a prosthetic graft connecting the RA to the PLSVC, with subsequent ligation of the PLSVC's inferior segment. These grafts can be routed anterior to the ascending aorta, within the transverse sinus, or posterior to the heart. Alternatively, some techniques advocate for PLSVC anastomosis to the right SVC using a prosthetic conduit. In contrast, our novel technique offers a technically simpler approach. It requires minimal modification to the standard cardiectomy procedure, avoids any prolongation of CPB duration, and eliminates the need for prosthetic materials altogether. This allows for a standard orthotopic HTx with a bicaval or biatrial anastomosis to be performed even if the PLSVC is identified intraoperatively. Postoperative echocardiography will always reveal 2 distinct CS: a large native one and a smaller originating from the donor heart. Although some existing methods share similarities with our approach, they necessitate meticulous isolation of the PLSVC, CS, and the LA cuff, followed by ligation of all coronary venous tributaries.^4^ This approach, however, is considerably more demanding in terms of technical skill and surgical time.

## Conclusions

Our technique of preservation of recipient CS and in situ routing of PLSVC during HTx in patients with PLSVC and a roofed coronary sinus is safe, simple, and easily reproducible. In addition, it avoids the need of prosthetic materials.

## Conflict of Interest Statement

The authors reported no conflicts of interest.

The *Journal* policy requires editors and reviewers to disclose conflicts of interest and to decline handling or reviewing manuscripts for which they may have a conflict of interest. The editors and reviewers of this article have no conflicts of interest.

## Supplementary Data

Video 1 Short intraoperative clip showing the persistent left superior vena cava draining through the roofed coronary sinus to the right atrium. Video available at: https://www.jtcvs.org/article/S2666-2507(24)00326-2/fulltext.

Video 2 Postoperative transesophageal echocardiogram revealed the presence of 2 distinct coronary sinuses. A, Donor's coronary sinus; B, recipient's coronary sinus draining persistent left superior vena cava (PLSVC). Video available at: https://www.jtcvs.org/article/S2666-2507(24)00326-2/fulltext.
