# Supplementary figures and images for: Heart transplant for a patient with left superior vena cava: Case report and surgical technique
Source: JTCVS Tech. 2024 Jul 30;27:132–4. doi: 10.1016/j.xjtc.2024.07.015 (PMC11518945; doi:10.1016/j.xjtc.2024.07.015)

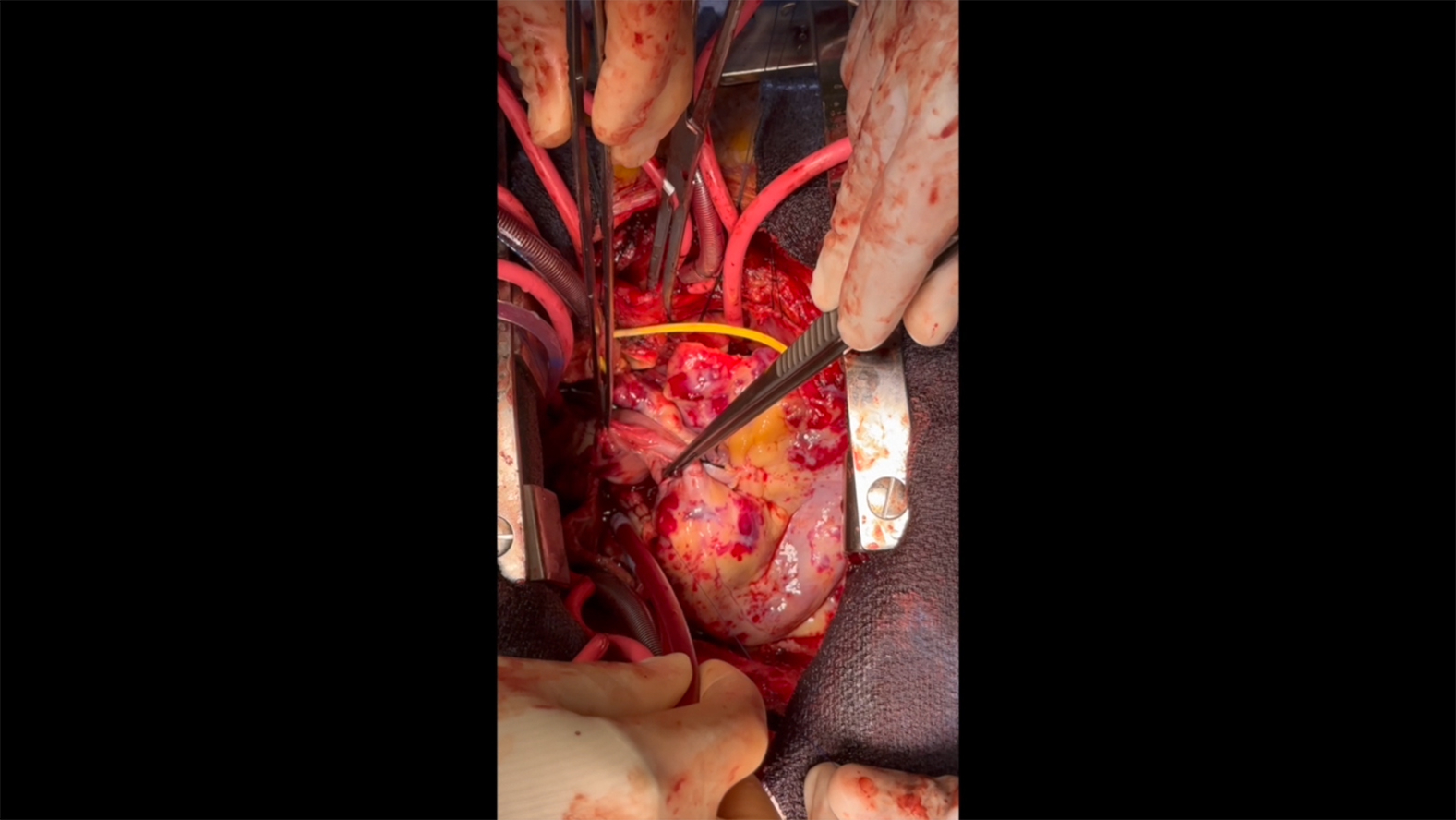

Supplement: Video 1 — Short intraoperative clip showing the persistent left superior vena cava draining through the roofed coronary sinus to the right atrium. Video available at: https://www.jtcvs.org/article/S2666-2507(24)00326-2/fulltext. [file fx2.jpg]

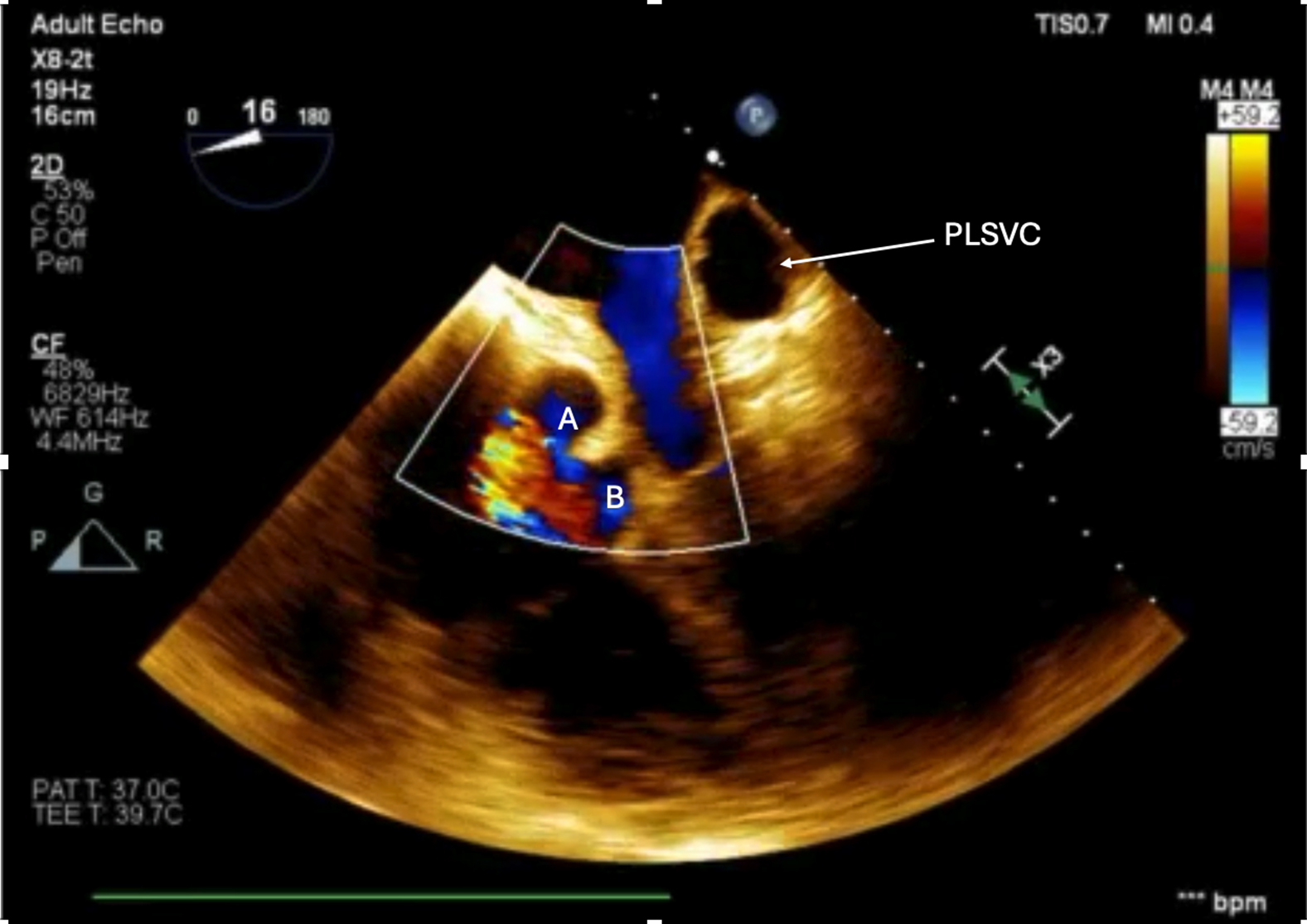

Supplement: Video 2 — Postoperative transesophageal echocardiogram revealed the presence of 2 distinct coronary sinuses. A, Donor's coronary sinus; B, recipient's coronary sinus draining persistent left superior vena cava (PLSVC). Video available at: https://www.jtcvs.org/article/S2666-2507(24)00326-2/fulltext. [file fx3.jpg]
